# Trends and Inpatient Outcomes of Venous Thromboembolism-Related Admissions in Patients with Philadelphia-Negative Myeloproliferative Neoplasms

**DOI:** 10.1055/s-0039-1692988

**Published:** 2019-07-17

**Authors:** Vatsala Katiyar, Alok Uprety, Andres Mendez-Hernandez, Harry E. Fuentes, Xavier A. Andrade, Maryam Zia

**Affiliations:** 1 Department of Medicine, John H. Stroger Jr. Hospital of Cook County, Chicago, Illinois, United States; 2 Department of Hematology and Oncology, Mayo Clinic, Rochester, Minnesota, United States; 3 Department of Hematology and Oncology, John H. Stroger Jr. Hospital of Cook County, Chicago, Illinois, United States

**Keywords:** venous thromboembolism, myeloproliferative neoplasm, polycythemia vera, essential thrombocytosis, primary myelofibrosis

## Abstract

**Background** Patients with Philadelphia-negative myeloproliferative neoplasms (MPNs), including polycythemia vera (PV), essential thrombocytosis (ET), and primary myelofibrosis (MF), have a significant risk of venous thromboembolism (VTE). We aim to determine the trends in annual rates of VTE-related admissions, associated cost, length of stay (LOS), and in-hospital mortality in patients with MPN.

**Methods** We identified patients with PV, ET, and MF from the Nationwide Inpatient Sample (NIS) database from 2006 to 2014 using ICD-9CM coding. Hospitalizations where VTE was among the top-three diagnoses were considered VTE-related. We compared in-hospital outcomes between VTE and non-VTE hospitalizations using chi-square and Mann–Whitney *U* -test and used linear regression for trend analysis.

**Results** We identified 1,046,666 admissions with a diagnosis of MPN. Patients were predominantly white (65.6%), females (52.7%), with a median age of 66 years (range: 18–108). The predominant MPN was ET (54%). There was no difference in in-hospital mortality between groups (VTE: 3.4% vs. non-VTE: 3.2%; *p* = 0.12); however, VTE admissions had a longer LOS (median: 6 vs. 5 days; *p* < 0.01) and higher cost (median: VTE US$32,239 vs. 28,403; *p* ≤ 0.01).

The annual rate of VTE admissions decreased over time (2006: 3.94% vs. 2014: 2.43%; *p* ≤ 0.01), compared with non-VTE–related admissions.

**Conclusion** In our study, VTE-related admissions had similar in-hospital mortality as compared with non-VTE–related admissions. The rates of hospitalizations due to VTE have decreased over time but are associated with a higher cost and LOS. Newer risk assessment tools may assist in preventing VTE in high-risk patients and optimizing resource utilization.

## Introduction

Philadelphia-negative myeloproliferative neoplasms (MPNs) are highly prevalent hematological malignancies that include polycythemia vera (PV), essential thrombocytosis (ET), and primary myelofibrosis (PMF). 1 2 These malignancies have been associated with a higher risk of venous thromboembolism (VTE) compared with the general population, leading up to one-third of patients with MPN experiencing VTE at any point in their disease. 3 4 5 The higher incidence of VTE in patients with MPN has led to development of recommendations of risk-adapted strategies to decrease their thromboembolism rate. 6

Despite risk-adapted preventive strategies, VTE remains one of the leading causes of death and healthcare expenditure in patients with cancer. 7 8 9 For instance, in a recent systematic review by Grosse et al, VTE had an estimated incremental cost of $12,000 to 15,000 (2014 U.S. dollars [USD]) per patient for first-VTE survivors and complications were estimated to increase cumulative costs up to $18,000 to 23,000; adding up to a total annual cost of 7 to 10 billion USD to the U.S. healthcare system. 10 Similarly, Barco et al estimated that VTE has a total annual cost of €1.5 to 2.2 billion and hospital-associated cost of €1.0 to 1.5 billion in the European Union. 11

Although there is emerging information of overall VTE-related costs, there is paucity of data of MPN-associated VTE mortality and healthcare expenditure in hospitalized patients. In this study, we aim to study annual rates of VTE-related admissions, length of inpatient stay (LOS), its associated cost, and in-hospital mortality in patients with MPN.

## Methods

### Study Design and Data Source

We queried the National Inpatient Sample (NIS) databases from 2006 to 2014 and conducted a retrospective analysis of hospitalizations in patients with Philadelphia-negative MPN. The NIS is a national database administered by the Healthcare Cost and Utilization Project sponsored by the Agency of Healthcare Research and Quality (AHRQ). The NIS consists of a stratified sample of approximately 20% of discharges from all community hospitals in the United States and provides weighted discharges to allow for national estimates.

### Population

We identified patients aged 18 or above, admitted with a diagnosis of PV, ET, and MF using International Classification of Diseases-Ninth Edition-Clinical Modification (ICD-9-CM) coding. Patients with PV were identified using ICD-9CM codes 238.4; patients with ET were identified using code 238.71; and patients with PMF were identified using codes 238.76 and 289.83.

### Variables

We obtained patients' baseline characteristics using variables provided by the NIS databases. These included age, gender, race, and healthcare payer.

We defined VTE-related hospitalization as an admission that included a VTE event in the primary discharge diagnosis or the initial two secondary diagnoses. A VTE event was allocated using ICD-9-CM codes 415.11, 415.13, and 415.19 for pulmonary embolism; 451.11, 451.2, 451.81, 453.40, 453.41, and 453.42 for deep vein thrombosis (DVT) of lower extremity; 453.0 for Budd-Chiari syndrome (BCS); and 451.19, 451.83, 451.84, 451.89, 451.9, 452.0, 453.2, 453.3, 453.8, 453.82, 453.83, 453.84, 453.85, 453.86, 453.87, 453.88, 453.89, and 453.90 for other types of VTE. Superficial vein thromboses were excluded.

Hospitalization costs were obtained using the total charges reported by hospitals to the NIS database. LOS in days was coded as a continuous variable. In-hospital mortality was coded as a binary variable coded from the discharge disposition of patient reported to the NIS database.

### Outcomes

Our primary outcomes included inpatient mortality, hospitalization cost, and LOS of all VTE-related admissions. In addition, we aimed to measure the trends of the annual rates of VTE-related admissions, cost of hospitalization, LOS, and in-patient mortality.

### Statistical Analysis

We used descriptive statistics to summarize the baseline characteristics of the cohort and subgroups. Continuous variables are expressed as mean and standard deviation (SD) or median and interquartile range (IQR) as appropriate. Categorical variables are expressed as absolute numbers and percentages.

National estimates for the annual rates of VTE-related hospitalizations were made using weights provided by the NIS database. We used simple linear regression to analyze the trends of annual rates of VTE-related hospitalizations and inpatient mortality. We compared the trends in annual cost of hospitalization and LOS between groups using Mann–Whitney *U* -test. All statistical analyses were performed using SPSS version 22.0 (IBM Corp., Armonk, NY).

## Results

We identified 1,046,666 admissions for patients with Philadelphia-negative MPNs from 2006 to 2014. Patients had a median age of 66 years (IQR: 26 years) and were predominantly Caucasians ( *n* = 686,104; 65.6%) and female ( *n* = 552,016; 52.7%). The most common MPN type was ET ( *n* = 565,644; 54%), followed by PV ( *n* = 313,588; 30%) and PMF ( *n* = 167,434; 16%).

Patients were predominantly over 60 years of age in all the three subgroups: 54.1, 62.9, and 89.9% in ET, PV, and PMF, respectively. Patients had similar demographic and health insurance payer among all MPN types as detailed in Tables 1 and 2 .

**Table 1:** Demographic characteristics of cohort by type of hospitalization

Characteristics		VTE hospitalization	Non-VTE hospitalization
		*n* = 39,124	*n* = 1,007,542
Age	≥60 y— *n* (%)	23,084 (59)	622,265 (61.7)
Gender	Female— *n* (%)	21,230 (54.3)	530,786 (52.7)
Race	White— *n* (%)	25,953 (75.4)	660,151 (73.6)
	African American— *n* (%)	4,802 (13.9)	121,856 (13.6)
	Hispanic— *n* (%)	2,222 (6.5)	68,849 (7.7)
	Asian or Pacific Islander— *n* (%)	385 (1.1)	18,931 (2.1)
	Native American— *n* (%)	132 (0.4)	4,878 (0.5)
	Other— *n* (%)	944 (2.7)	22,755 (2.5)
Insurance	Medicare— *n* (%)	20,310 (51.9)	568,699 (56.6)
	Medicaid— *n* (%)	3,948 (10.1)	111,371 (11.1)
	Private insurance— *n* (%)	10,967 (28.0)	233,965 (23.3)
	Self-pay— *n* (%)	2,288 (5.9)	56,268 (5.6)
	No charge— *n* (%)	300 (0.8)	6,278 (0.6)
	Other— *n* (%)	1,261 (3.2)	29,080 (2.9)
Tumor type	PV— *n* (%)	13,057 (33.3)	300,531 (29.8)
	ET— *n* (%)	20,520 (52.4)	545,124 (54.1)
	PMF— *n* (%)	5,548 (14.1)	161,886 (16.1)

Abbreviations: ET, essential thrombocythemia; MPN, myeloproliferative neoplasms; PMF, primary myelofibrosis; PV, polycythemia vera; VTE, venous thromboembolism.

**Table 2:** Demographic characteristics of cohort by tumor type

Characteristics		MPN type		
		PV	ET	PMF
		*n* = 313,588	*n* = 565,644	*n* = 167,434
Age	≥60 y— *n* (%)	197,156 (62.9)	305,972 (54.1)	142,220 (89.9%)
Gender	Male— *n* (%)	182,482 (58.2)	227,978 (40.3)	84,149 (50.3)
Race	White— *n* (%)	222,551 (81.1)	343,926 (67.9)	119,627 (79.5)
	African American— *n* (%)	22,580 (8.2)	89,569 (17.7)	14,508 (9.6)
	Hispanic— *n* (%)	16,839 (6.1)	45,360 (8.9)	8,873 (5.9)
	Asian or Pacific Islander— *n* (%)	4,758 (1.7)	11,000 (2.2)	3,558 (2.4)
	Native American— *n* (%)	1,369 (0.5)	3,165 (0.6)	476 (0.3)
	Other— *n* (%)	6,388 (2.3)	13,809 (2.7)	3,501 (2.3)
Insurance	Medicare— *n* (%)	180,976 (57.8)	281,991 (49.9)	126,041 (75.4)
	Medicaid— *n* (%)	27,920 (8.9)	79,649 (14.1)	7,750 (4.6)
	Private insurance— *n* (%)	74,685 (23.9)	141,961 (25.1)	28,285 (16.9)
	Self-pay— *n* (%)	18,680 (6.0)	37,569 (6.7)	2,308 (1.4)
	No charge— *n* (%)	1,954 (0.6)	4,353 (0.8)	271 (0.2)
	Other— *n* (%)	8,737 (2.8)	19,092 (3.4)	2,512 (1.5)
Type of hospitalization	Non-VTE related— *n* (%)	301,125 (95.8)	545,757 (96.5)	162,082 (96.8)
	VTE related— *n* (%)	13,057 (4.2)	20,520 (3.5)	5,548 (3.2)

Abbreviations: ET, essential thrombocythemia; MPN, myeloproliferative neoplasms; PMF, primary myelofibrosis; PV, polycythemia vera; VTE, venous thromboembolism.

We abstracted 39,124 (3.7%) VTE-related admissions during the study period. VTE hospitalizations were higher in patients with PV ( *n* = 13,057, 4.2%) followed by ET ( *n* = 20,520; 3.5%) and PMF ( *n* = 5,548, 3.2%; Table 2 ).

In our cohort, the overall mortality rate was similar in patients with VTE-related hospitalization compared with non-VTE admissions (3.3 vs. 3.2%; *p* = 0.646). Nonetheless, in MPN subgroups, patients with PV had a mortality in VTE-related admissions compared with non-VTE–related admissions (3.5 vs. 3.1%; *p* = 0.009). Mortality was similar among VTE- and non-VTE admissions for patients with ET (2.5 vs. 2.3%; *p* = 0.053) and PMF (5.6 vs. 6.6%; *p* = 0.002). The case-fatality rate for VTE-related admissions was 0.12% ( Table 3 ).

**Table 3:** Outcomes by VTE event in the entire cohort

Outcomes	Non-VTE hospitalization	VTE hospitalization	*p* -Value
Mortality— *n* (%)	32,617 (3.2)	1,283 (3.3)	0.646
Total cost of hospitalization (USD)—median (IQR)	28,426 (42,572)	31,549 (46,097)	0.001
Total length of stay (days)—median (IQR)	5 (5)	6 (6)	<0.001

Abbreviations: IQR, interquartile range; VTE, venous thromboembolism.

Overall, patients with a VTE-related hospitalization had a higher median cost of hospitalization (USD 31,549 vs. 28,426; *p* = 0.001) and longer LOS (6 vs. 5 days; *p* ≤ 0.001) compared with non-VTE hospitalizations. Among VTE admissions, patient with ET had the highest cost (median: USD 35,703 [IQR = 53,703]) and LOS (median: 6 days [IQR = 7]) compared with PV (median: USD 25,745; median LOS: 5 days [IQR = 4]) and PMF (median: USD 33,213; median LOS: 6 days [IQR = 7]; Tables 3 and 4 ).

**Table 4:** Outcomes of VTE hospitalizations by tumor type

Outcomes	PV		ET		PMF		*p* -Value		
	Non-VTE	VTE	Non-VTE	VTE	Non-VTE	VTE	PV vs. ET (VTE vs. VTE)	PV vs. PMF (VTE vs. VTE)	ET vs. PMF (VTE vs. VTE)
Hospitalization	301,125 (95.8)	13,057 (4.2)	545,757 (96.5)	20,520 (3.5)	162,082 (96.8)	5,548 (3.2)	<0.001	<0.001	<0.001
Mortality— *n* (%)	9,290 (3.1)	457 (3.5)	12,633 (2.3)	518 (2.5)	10,694 (6.6)	308 (5.6)	<0.001	<0.001	<0.001
Total cost of hospitalization (USD)—median (IQR)	23,542 (32,083)	25,745 (34,962)	31,729 (48,947)	35,805 (53,703)	28,942 (43,096)	33,213 (47,337)	<0.001	<0.001	0.115
Total length of stay (days)—median (IQR)	4 (4)	5 (4)	5 (7)	6 (7)	5 (5)	6 (7)	<0.001	<0.001	0.300

Abbreviations: ET, essential thrombocythemia; PMF, primary myelofibrosis; PV, polycythemia vera; VTE, venous thromboembolism.

Acute DVT was the most common VTE event in patients with PV ( *n* = 5,731, 43.9%) and PMF ( *n* = 1824, 32.9%), and PE was more common in patients with ET ( *n* = 8,733, 42.6%). BCS was the third most common VTE event in all the three MPNs (PV: 4.4%, ET: 1.2%, PMF: 7.7%; Table 5 ).

**Table 5:** VTE events per MPN type

VTE subtype	PV	ET	PMF	*p* -Value		
				PV vs. ET	PV vs. PMF	ET vs. PMF
Acute DVT— *n* (%)	5,731 (43.9%)	7,490 (36.5%)	1,824 (32.9%)	<0.001	<0.001	0.003
Pulmonary embolism— *n* (%)	4,586 (35.1%)	8,733 (42.6%)	1,724 (31.1%)	<0.001	<0.001	<0.001
Bud-Chiari syndrome— *n* (%)	568 (4.4%)	250 (1.2%)	428 (7.7%)	<0.001	0.007	<0.001
Other— *n* (%)	2,172 (16.6%)	4,047 (19.7%)	1,572 (28.3%)	<0.001	<0.001	<0.001

Abbreviations: DVT, deep vein thrombosis; ET, essential thrombocythemia; MPN, myeloproliferative neoplasm; PMF, primary myelofibrosis; PV, polycythemia vera; VTE, venous thromboembolism.

**Fig. 1:**
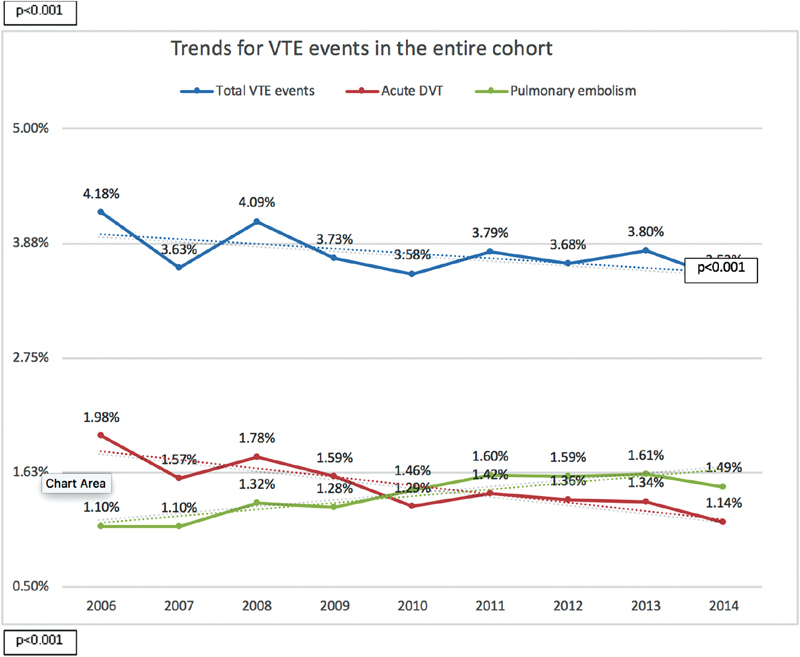
Trends for VTE events in the entire cohort. VTE, venous thromboembolism.

During the study period, there was a trend toward a decreased total VTE-related admissions (2006 = 4.18% vs. 2014 = 3.53%; *p* < 0.001) and increased mortality (2006 = 2.77% vs. 2014 = 4.94%; *p* < 0.001) and costs per hospitalization (2006 = USD 22,702 vs. 2014 = USD 37,100; *p* < 0.001). Notably, there was a trend toward an increased yearly rate of PE-related admissions (2006 = 1.1% vs. 2014 = 1.49%; *p* < 0.001), PE-related in-hospital mortality (2006 = 6.21% vs. 2014 = 7.96%; *p* < 0.001), and PE hospitalization costs (2006 = USD 27,027 vs. 2014 = USD 42,959; *p* ≤ 0.001; Figs. 1 2 3 ).

**Fig. 2:**
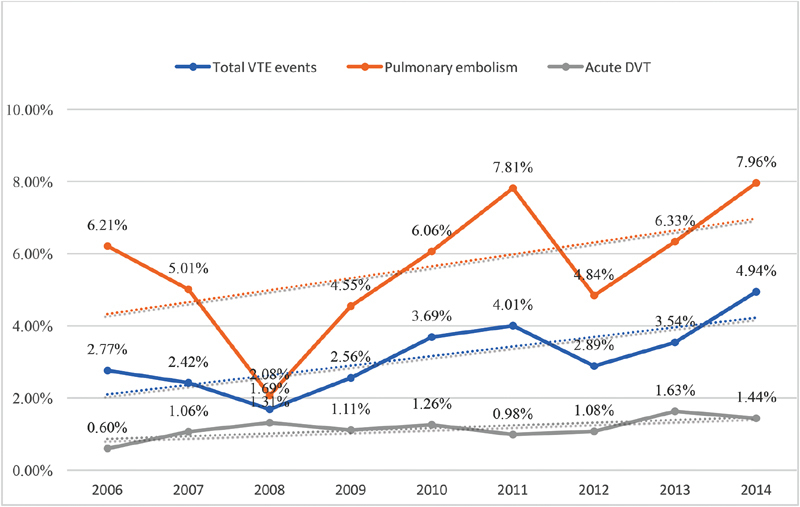
Trends of in-hospital mortality of VTE-related hospitalizations. DVT, deep vein thrombosis; VTE, venous thromboembolism.

**Fig. 3:**
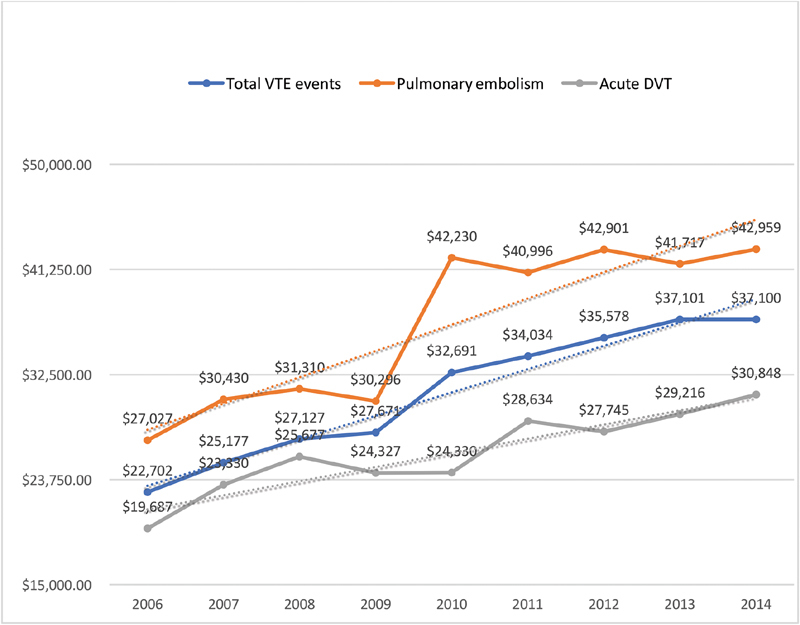
Trends in total costs per VTE admission in U.S. dollars. DVT, deep vein thrombosis; VTE, venous thromboembolism.

## Discussion

This retrospective analysis was designed to examine the annual trends in VTE-related hospitalizations among patients with Philadelphia-negative MPNs and their associated cost, LOS, and inpatient mortality.

We studied hospitalizations during the period of 2006 to 2014 in our cohort of MPN patients. From a total of 1,046,666 admissions, we abstracted 39,124 (3.7%) VTE-related admissions. ET was the most frequently identified MPN from all VTE hospitalizations (20,520; 52.4%) followed by PV (13,057; 33.3%) and PMF (5,548; 14.1%).

The majority of patients in our cohort were 60 years or older, particularly in the PMF subgroup (89.9%). Age is a well-recognized risk factor for thrombosis in patients with MPN. For instance, the European Collaboration on Low-dose Aspirin in PV (ECLAP) study demonstrated that age over 65 years led to increased risk of a thromboembolic event in PV patients. 12 Similarly, elderly patients with ET have an increased risk for VTE. Consequently, well-validated risk stratification models like the International Prognostic Scoring for Thrombosis in ET patients (IPSET) criteria incorporate age over 60 years as a criterion for prediction of thrombotic events. 13

When we analyzed VTE hospitalizations by MPN tumor types, we observed a higher rate of VTE admissions in patients with PV compared with ET and PMF (4.2 vs. 3.5 vs. 3.2%; *p* ≤ 0.001). The higher incidence of VTE in patients with PV compared with other MPN has been previously and extensively described. 14 It is plausible that the higher incidence of VTE in patients with PV would lead to a higher rate of VTE-related hospitalizations when compared with other MPNs as observed in our study population.

When examining VTE subtypes in our cohort, DVT was more common in patients with PV (43.9% of VTE events in PV group), while PE was more common in ET patients (42.6% of VTE events within ET group). Specific risk factors for development of PE in MPN patient have not been previously described and remain an area of investigation.

In our cohort, PMF patients had the highest rate of BCS compared with other MPN (PMF: 7.7% vs. PV: 4.4% vs. ET: 1.2%; *p* ≤ 0.001). Although the association of MPN to BCS is well known, PMF has been described as having a lower prevalence among BCS patients. For instance, in a recent meta-analysis of 1,062 patients with BCS, Smalberg et al reported the prevalence of PMF to be lowest (6.7%; 95% confidence interval [CI]: 32.3–49.5%) when compared with PV (52.9%; 95% CI: 42.2–63.4%) and ET (24.6%; 95% CI: 18–32.2%). 15 The higher incidence of BCS in our cohort could be caused by a selection of a high-risk group of PMF patients with other concurrent tumor- and patient-dependent VTE risk factors not included in the NIS database like JAK2V617F mutation burden. 16 Furthermore, the higher rate of thrombocytopenia in PMF may have led to higher VTE hospitalization rate due to bleeding complications during anticoagulation therapy.

### Comparison of In-Patient Mortality Rates, Cost, and Length-of-Stay in MPN Patients with VTE Hospitalizations

In our cohort, mortality was similar in VTE versus non-VTE groups (3.3 vs. 3.2; *p* = 0.646). In contrast, cancer-associated thrombosis has been linked to a poorer prognosis in hospitalized patients. For instance, in a cohort of 3,146,388 hospitalized patients with cancer, Lyman et al demonstrated a higher in-hospital mortality for patients with cancer-related VTE (15 vs. 5.5%) compared with non-cancer–related VTE. 17 This difference may be explained by a more indolent course of MPNs compared with solid tumors included in other descriptive studies. Moreover, the presence of less severe forms of VTE (e.g., upper extremity DVT and lower extremity DVT) in our cohort could have contributed to comparable rates of mortality in the VTE- and non-VTE–related hospitalization groups.

In our study population, VTE-related admissions in MPN patients were associated with a higher cost (USD 31,549 vs. 28,426; *p* = 0.001) and a longer LOS (6 vs. 5 days, *p* ≤ 0.001), similar to findings of other descriptive studies. 18 The higher costs are commonly due not only to anticoagulation therapy but also to diagnostic imaging studies and bleeding complications occurring in approximately 8% of all MPN patients. 19

### Trends of Yearly Rates of VTE Hospitalizations in Patients with MPN

We observed a decline in VTE-related admissions of MPN patients over the study period (2006: 3.94% vs. 2014: 2.43%; *p* < 0.001). This is potentially explained by a more widespread use of risk-adapted strategies to decrease the risk of VTE in high-risk patients using antiplatelet drugs and cytoreductive agents. 20 21 Moreover, changes in practice regarding outpatient management of DVT could have potentially reduced DVT-related admissions throughout the study period.

Interestingly, we observed a trend toward an increase in incidence of PE-related admissions during the study period (2006: 1.1% vs. 2014: 1.49%, *p* < 0.01). This change may reflect the increased detection of PE since the widespread use of computed tomography angiography for diagnosis. 22 However, it is still possible that there are other biological or environmental factors that predispose MPN patients to develop PE, particularly patients with ET.

The major strength of this study is that we used an NIS database. This allowed us to abstract a large cohort of patients with MPN and thus obtain robust estimations regarding outcomes of patients hospitalized for VTE. Limitations of the study include those inherent to retrospective studies and the absence of a marker of severity of VTE coded in NIS database, precluding our study from analyzing the impact of VTE severity on overall mortality, cost, and LOS in VTE hospitalizations. In addition, other factors like duration of MPN disease before occurrence of VTE, choice of anticoagulation, or use of prior cytoreductive therapy could not be obtained from the NIS database.

One limitation of our study is the inability to estimate the costs of care after hospitalization. It is unknown if the cost of care will eventually be similar in VTE- and non-VTE–related admissions after hospitalization. However, the focus of our study was to examine in-hospital outcomes to delineate strategies that would allow for the prevention of VTE in patients with MPN which, in turn, would improve resource allocation for the care of this patient population.

Another limitation of our study is that the exact timing of VTE in the NIS database cannot be determined. Nonetheless, we accounted for this limitation by selecting patients with a VTE event on the principal or the top-two secondary diagnosis, which can be reliably assumed to be acute and a main concern during hospitalization. This allowed an accurate selection of patients with a VTE-related hospitalization and enabled us to provide an estimate of their outcomes.

In conclusion, VTE-related admissions in patients with MPN were associated with higher costs and prolonged hospitalization. Furthermore, there is a decline in VTE-related admissions of MPN patients, although yearly rates of PE-related admissions have increased from 2006 to 2014, regardless of the type of MPNs. Further refining our risk-stratification strategy to include clinical, cytogenetic, and biological data may assist in preventing VTE in high-risk patients and decrease VTE-related mortality and cost burden in patients with MPNs.
